# CORRELATION BETWEEN CONTROLLED-PRESSURE PROVOCATIVE TEST DURATION AND ELECTRODIAGNOSTIC SEVERITY IN CARPAL TUNNEL SYNDROME

**DOI:** 10.2340/jrm.v58.44203

**Published:** 2026-01-27

**Authors:** Apiphan IAMCHAIMONGKOL, Khanin LEEAREEKUN, Waree CHIRA-ADISAI, Apisara KEESUKPHAN

**Affiliations:** Department of Rehabilitation Medicine, Faculty of Medicine Ramathibodi Hospital, Mahidol University, Thailand

**Keywords:** carpal tunnel syndrome, electrodiagnosis, median neuropathy, pressure, provocative

## Abstract

**Objective:**

To assess the correlation between controlled-pressure provocative test duration and electrodiagnostic severity in carpal tunnel syndrome.

**Design:**

Cross-sectional correlational study.

**Patients:**

Patients with clinical symptoms consistent with carpal tunnel syndrome were recruited from the electrodiagnosis clinic of the Department of Rehabilitation Medicine, Faculty of Medicine Ramathibodi Hospital, Mahidol University, Bangkok, Thailand, between September 2023 and July 2024.

**Methods:**

Patients underwent electrodiagnostic studies, and the severity of carpal tunnel syndrome was classified as mild, moderate, or severe according to the 2011 criteria of the American Association of Neuromuscular & Electrodiagnostic Medicine (AANEM). Each participant subsequently received a controlled-pressure provocative test. The time to symptom provocation was recorded, with a maximum duration of 30 s. Spearman’s rank correlation was used to assess the association between test duration and electrodiagnostic severity.

**Results:**

In 124 hands, 31, 45, and 48 hands were categorized by the degree of electrodiagnostic severity as mild, moderate and severe, respectively. There was no correlation (ρ = –0.16, *p*-value = 0.074) between controlled-pressure provocative test duration and electrodiagnostic severity.

**Conclusions:**

There was no correlation between controlled-pressure provocative test duration and electrodiagnostic severity in patient with carpal tunnel syndrome.

## LAY ABSTRACT

Carpal tunnel syndrome is a condition in which the median nerve in the wrist is compressed, causing symptoms like numbness and tingling. This study investigated whether the time it takes for provoked symptoms during a controlled-pressure Carpal Compression Test is related to the severity of nerve damage, classified by electrodiagnosis study in 124 hands. The results showed no significant relationship between the time to symptom appearance and the severity of carpal tunnel syndrome. These findings suggest that the duration of the wrist pressure test may not be a reliable indicator of the severity of nerve damage in carpal tunnel syndrome.

Carpal tunnel syndrome (CTS), or median neuropathy at the wrist, is the most common condition involving nerve compression. Patients typically present with numbness in the thumb, index, middle, or ring fingers (1–3). Diagnosis of CTS primarily relies on clinical symptoms, with electrodiagnostic studies used to confirm the diagnosis and assess severity (1–3). Several physical examination methods can provoke symptoms to aid diagnosis, such as Phalen’s Test, Modified Phalen’s Test, and Carpal Compression Test, all demonstrating high sensitivity and specificity (3–5). These tests provoke symptoms by increasing pressure in the carpal tunnel, causing ischaemia in the median nerve and symptom reproduction. The principle of pressure-provocation testing is based on the concept that chronically compressed nerves are more sensitive to external pressure (6–10).

While their diagnostic sensitivity and specificity have been well established, it remains unclear whether the time to symptom provocation – i.e., the duration from test initiation to a positive response – is associated with the severity of CTS as determined by electrodiagnostic studies. Few studies have examined the relationship between the duration of a positive Phalen’s Test and electrodiagnostic findings in patients with CTS. Previous research has shown that shorter Phalen’s Test durations are associated with higher specificity and positive predictive value for detecting abnormal electrodiagnostic results; however, the correlation between Phalen’s Test duration and the severity of electrodiagnostic findings was weak (11). Another study reported that Phalen’s Test yielded similar results in cases with both mild and severe electrodiagnostic severity (12).

Beyond duration-based findings, additional studies have evaluated whether test positivity itself, rather than duration, correlates with CTS severity. Izadi et al. (13) found no relationship between a positive result on Phalen’s Test, Reverse Phalen’s Test, Tinel’s sign, or the manual Carpal Compression Test, and CTS severity.

The Carpal Compression Test was developed by Durkan in 1991 (14). In this test, the examiner uses both thumbs to apply direct pressure over the carpal tunnel for 30 s. A positive result is indicated by the reproduction of paraesthesia in the median nerve distribution. However, no previous studies have examined whether the time to symptom reproduction during the Carpal Compression Test correlates with electrodiagnostic severity. Specifically, the relationship between duration of symptom onset during the test and CTS severity has not been explored. Furthermore, in clinical practice, the applied pressure during the Carpal Compression Test is typically unmeasured, leading to variability in the test. To address this limitation, the present study used a controlled-pressure provocative test, which involves wrapping a sphygmomanometer cuff around the wrist, inflating the cuff, and applying additional finger pressure until the gauge reads 150 mmHg (15).

Therefore, this study aims to investigate whether the duration of the controlled-pressure provocative test, measured from the start of the test to the point of a positive result, is correlated with the severity of carpal tunnel syndrome as determined by electrodiagnostic studies.

## METHODS

### Study design

This study was a cross-sectional correlational study. Participants with CTS were recruited through the electrodiagnosis clinic of the Department of Rehabilitation Medicine, Faculty of Medicine Ramathibodi Hospital, Mahidol University, Bangkok, Thailand, from September 2023 to July 2024. This trial was approved by the Committee on Human Rights Related to Research involving Human Subjects of the Faculty of Medicine Ramathibodi Hospital, Mahidol University, Bangkok, Thailand (MURA2023/643).

### Study populations

The inclusion criteria were patients presenting with clinical symptoms consistent with CTS, typically manifesting as numbness in the thumb, index, or middle finger (1, 3), confirmed by electrodiagnosis. Participants must also be over age 18 and willing to join the study. Patients were excluded from the study if they had median nerve pathology caused by conditions other than idiopathic CTS, such as infections, tumour, or traumatic injuries. Those who had peripheral neuropathy, a history of carpal tunnel release surgery, cervical radiculopathy, or brachial plexopathy were also excluded from participation.

### Sample size

To examine the relationship between duration of provocation of the controlled-pressure provocative test and the severity assessed by electrodiagnostic evaluation, with an expected correlation coefficient of 0.25, a significance level of 0.05, and a statistical power of 80%, the required sample size is 123 hands.

### Study procedures

**Fig. 1:**
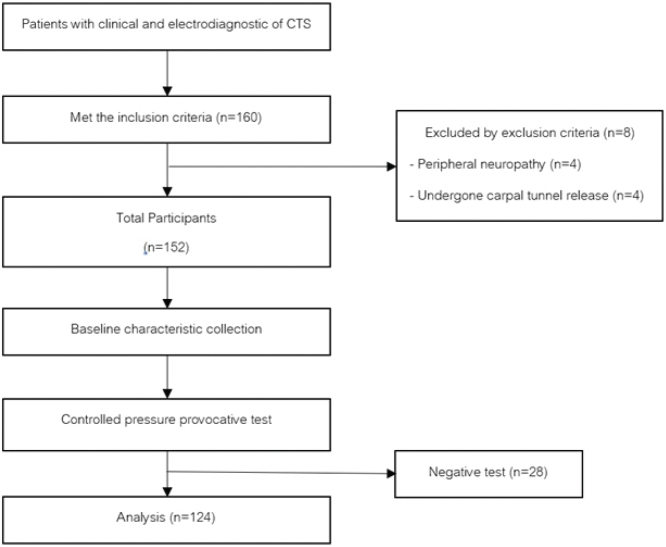
Flowchart of the study.

The researchers recruited participants through an invitation process involving project promotion and verbal communication. The flow of the study is shown in **Fig. 1**. The researchers explained the study details and procedures to the participants, who then signed informed consent forms. Baseline data, including age, sex, weight, height, body mass index (BMI), occupation, symptom duration, and severity based on electrodiagnostic evaluation, were collected.

A physiatrist performed the electrodiagnostic study. The study was conducted using the Cadwell Sierra Summit® system (Cadwell Industries Inc, Kennewick, WA, USA). The electrodiagnostic technique was applied according to the 2011 practice recommendations for CTS provided by the American Association of Neuromuscular & Electrodiagnostic Medicine (AANEM) (16). Severity was categorized into 3 groups according to the AANEM guidelines: mild (prolonged sensory latency with normal motor studies and no axonal loss), moderate (abnormal sensory latency with prolonged motor distal latency and no axonal loss), or severe (any nerve conduction abnormalities with axonal loss, identified through low or absent sensory/motor action potentials or needle electromyography changes) (17).

**Fig. 2:**
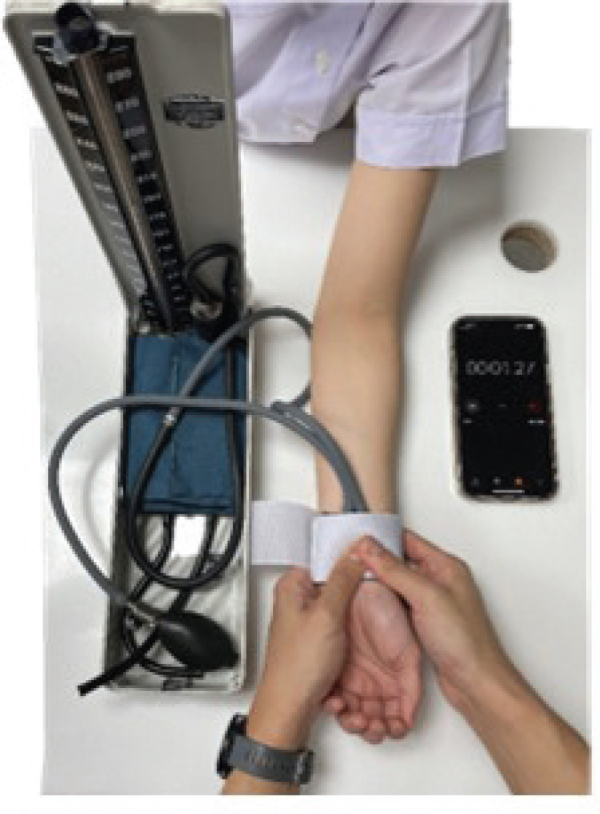
Controlled-pressure provocative test.

The participants then underwent a controlled-pressure provocative test, as shown in Fig. 2, to induce symptoms. This test used the cuff of a sphygmomanometer wrapped around the wrist of the hand being assessed. The researcher inflated the cuff to a pressure of 50 mmHg and then pressed on the cuff until the sphygmomanometer displayed a pressure of 150 mmHg. Timing began when the participant reported provocation of the symptoms, at which point the time was recorded. If no increased numbness occurred within 30 s, the test was stopped, and the result was recorded as negative.

### Statistical analysis

The data were analysed using STATA version 18 (StataCorp, College Station, TX, USA). Descriptive statistics were used to present categorical data as numbers (percentages), normally distributed continuous data as mean (SD), and non-normally distributed continuous data as median (IQR). Differences in baseline characteristics and time to positive response in the controlled-pressure provocative test were compared using Pearson’s χ^2^ test for categorical data, one-way ANOVA for normally distributed continuous data, and the Kruskal–Wallis test for non-normally distributed continuous data.

Spearman’s rank correlation coefficient was used to analyse associations in non-normally distributed data, presented as the correlation coefficient (ρ). A *p*-value of < 0.05 was considered statistically significant.

## RESULTS

A total of 160 hands met the inclusion criteria. Eight hands were excluded based on the exclusion criteria: 4 due to peripheral neuropathy and 4 due to a history of carpal tunnel release. The remaining 152 hands underwent the controlled-pressure provocative test, with 28 yielding negative results. Ultimately, 124 hands from 78 patients were included in the final analysis.

Participants with CTS were classified into 3 groups based on electrodiagnostic severity: mild (31 hands), moderate (45 hands), and severe (48 hands). A statistically significant difference in age was observed among the groups, with the severe group having the highest mean (SD) age of 64.67 (13.00) years, followed by the moderate and mild groups at 55.16 (12.07) and 54.29 (11.54) years, respectively. In contrast, no significant differences were found between the groups in terms of sex, body mass index (BMI), symptom duration, affected hand, or hand dominance. However, occupation significantly differed among the groups (*p* = 0.015), as indicated in Table I.

**Table I:** Demographic data of participants

Factor	Mild (*n* = 31)	Moderate (*n* = 45)	Severe (*n* = 48)	*p*-value
Age, year, mean (SD)	54.29 (11.54)	55.16 (12.07)	64.67 (13.00)	**< 0.001**
Sex, *n* (%)				0.413
Male	4 (12.90)	7 (15.56)	8 (16.67)	
Female	27 (87.10)	38 (84.44)	40 (83.33)	
BMI, mean (SD)	26.58 (5.31)	25.43 (3.30)	25.16 (3.76)	0.297
Duration of symptom, month, median (IQR)	6 (1.5,20)	12 (3,24)	6 (3,12)	0.218
Affected hand, *n* (%)				0.248
Right	6 (19.35)	3 (6.67)	9 (18.75)	
Left	6 (19.35)	5 (11.11)	8 (16.67)	
Bilateral	19 (61.30)	37 (82.22)	31 (64.58)	
Dominant hand, *n* (%)				0.229
Right	29 (93.55)	36 (80.00)	42 (87.50)	
Left	2 (6.45)	9 (20.00)	6 (12.50)	
Occupation, *n* (%)				0.015
Homemaker	16 (16.61)	12 (26.67)	23 (47.92)	
Janitor/Janitress	3 (9.68)	14 (31.11)	10 (20.83)	
Office worker	8 (25.81)	5 (11.11)	5 (10.42)	
Hospital worker	3 (9.68)	3 (6.67)	1 (2.08)	
Industrial worker	0 (0)	9 (20.00)	7 (14.58)	
Farmer	1 (3.23)	2 (4.44)	2 (4.17)	

Values shown in bold indicate statistically significant differences (*p*<0.05).

The median (IQR) times to symptom onset during the controlled-pressure provocative test across all severities was 15 s (10–23). Specifically, it was 13 s (8.5–23) in the severe group, 14 s (8–21) in the moderate group, and 19 s (13–24) in the mild group, as presented in Table II, with no statistically significant difference among groups (*p* = 0.069).

**Table II:** Time to positive pressure provocative test

Item	Mild (*n* = 31)	Moderate (*n* = 45)	Severe (*n* = 48)	*p*-value
Time to positive, s, median (IQR)	19 (13,24)	14 (8,21)	13 (8.5,23)	0.069

**Fig. 3:**
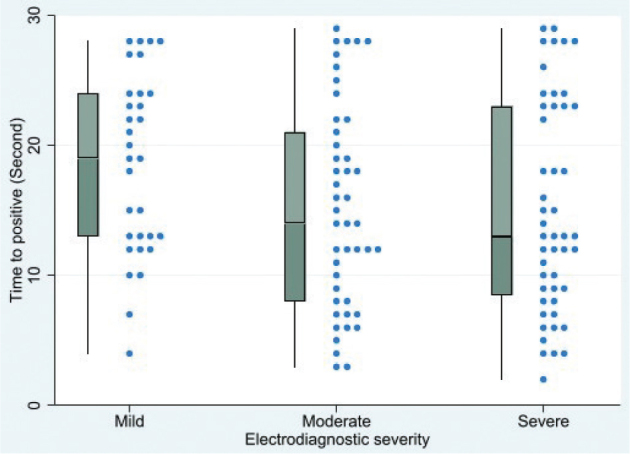
Box and dot plot illustrating the distribution of time to symptom onset during the controlled-pressure provocative test across 3 levels of electrodiagnostic severity in carpal tunnel syndrome. No significant correlation was found between test duration and severity (ρ = −0.16, *p* = 0.074).

No significant correlation was observed between the time to symptom onset during the controlled-pressure provocative test and the electrodiagnostic severity of carpal tunnel syndrome. The Spearman’s correlation coefficient was −0.16 (*p* = 0.074), as illustrated in Fig. 3.

## DISCUSSION

This study found no correlation between the time to symptom onset in the controlled-pressure provocative test and the severity of carpal tunnel syndrome. However, the time to symptom onset tended to be longer in the mild severity group compared with the severe group, although the difference was not statistically significant. The overall median (IQR) time to symptom onset in our study was 15 s (10–23). Compared with the study by Williams et al. (15) , which used the same controlled-pressure provocative method (150 mmHg), our study showed a longer time to symptom onset. Williams et al. reported a mean time of 9 s to positive response. This discrepancy may be due to differences in the study populations of patients with CTS. Notably, their study relied solely on clinical diagnosis without electrodiagnostic confirmation, whereas our study included participants with electrodiagnostically confirmed CTS severity, which may have influenced the comparability of findings.

The selection of 150 mmHg and a 30-s duration for the controlled-pressure provocative test in this study was based on previous literature demonstrating high diagnostic accuracy with this protocol. Williams et al. (15) compared pressure levels of 100 mmHg and 150 mmHg and found that 150 mmHg yielded a sensitivity of 100% and specificity of 98%, with an average symptom onset time of 9 s, while 100 mmHg showed a sensitivity of 98% and specificity of 100%, with an average onset time of 19 s. These findings suggest that higher pressure provokes symptoms more rapidly and with greater diagnostic consistency.

Duncan et al. (14) similarly used 150 mmHg applied via an atomizer bulb and both thumbs for 30 s in a Carpal Compression Test, reporting a sensitivity of 87% and specificity of 90%. These data collectively support the use of 150 mmHg for 30 s as a valid and reliable approach for symptom provocation in CTS assessment.

Our findings are consistent with prior studies showing that neither test duration of symptom provocation nor the presence of a positive provocative test reliably reflects electrodiagnostic severity (11–13, 18). Dabbagh et al. (19) found that the Carpal Compression Test was the most sensitive test (87%). It is confirmed that provocative tests remain useful for diagnosis, but their timing is unreliable for grading severity.

In our study, among the 28 hands with a negative controlled-pressure provocative test result, 23 hands (82.14%) were classified into the mild and moderate groups, and 5 hands (17.86%) were in the severe group. These results suggest that false-negative results occurred predominantly in less severe CTS. One possible explanation is that patients with mild or moderate CTS may have lower mechanosensitivity to externally applied pressure due to limited demyelination or axonal involvement, resulting in failure to reproduce typical symptoms within the 30-s testing window (8). Conversely, patients with severe CTS, who have more advanced pathophysiological changes, may exhibit heightened susceptibility to pressure provocation, thereby reducing the likelihood of a negative result.

Treatment decisions in CTS depend closely on severity. Mild and moderate cases are commonly managed conservatively, including splinting, lifestyle modification, and corticosteroid injection, with many patients responding well to non-surgical care (20, 21). In contrast, patients with severe CTS are significantly more likely to receive surgical decompression and tend to undergo surgery earlier than those with less severe disease (21). Therefore, grading severity remains essential for informing clinical decision-making, and the duration of a provocative test cannot replace electrodiagnostic evaluation.

Provocative manoeuvres remain clinically useful for diagnosing CTS. However, our findings indicate that the time to symptom provocation provides minimal additional diagnostic or prognostic value beyond a simple positive or negative result. The absence of a meaningful correlation between onset time and electrodiagnostic severity suggests that clinicians should not rely on timing as an indicator of disease severity. A recent study by Dabbagh et al. (22) suggested that clinicians should combine provocative manoeuvres with sensorimotor tests, hand diagrams, and diagnostic questionnaires to achieve better overall diagnostic accuracy rather than relying on individual clinical test.

Although this method utilized a sphygmomanometer cuff, it was not intended for routine clinical use but rather served as a standardized tool to provide quantitative information under controlled conditions. The aim was to minimize variability in pressure application and enhance reproducibility across participants (15). In clinical practice, however, direct thumb pressure over the carpal tunnel is commonly used, as seen in the manual Carpal Compression Test (14). This manual approach is more practical and feasible in real-world settings, although it may be subject to greater variability in applied pressure between examiners. Future studies could quantify examiner-dependent variability more formally by using repeated measurements across different examiners to evaluate inter-rater reliability, which would allow for a more objective assessment of how consistently the controlled-pressure technique is applied. However, our results show that the duration of symptom provocation does not correlate with CTS severity, indicating that this modification does not meaningfully add clinical value for severity grading or treatment planning.

The limitations of this study include the lack of blinding for the examining physician during data collection, which may introduce bias in the data-gathering process. However, the primary outcome of this study – the time to symptom onset – was based on patient-reported provocation of symptoms, which helps reduce examiner-dependent interpretation, although subtle influences such as examiner expectation cannot be entirely excluded. In addition, because only electrodiagnostically confirmed CTS cases were included, the study was unable to evaluate false-positive results or determine the specificity of the controlled-pressure provocative test. Including EMG-negative cases would be necessary to assess diagnostic accuracy and should be considered in future research. This study also did not explore the relationship between the duration of the controlled-pressure provocative test and clinical severity as well as prognosis, such as using the Boston Carpal Tunnel Syndrome Questionnaire (23) to assess severity. If this questionnaire had been used, it would have provided complementary information regarding symptom severity and functional impact. Previous research (10) on Phalen’s Test has shown that a positive result with a duration of less than 30 s is associated with a poorer prognosis and a higher likelihood of failure with conservative treatments. These findings suggest that the timing of symptom provocation may capture prognostically meaningful aspects of median nerve dysfunction. Prognosis may be more clinically relevant than severity grading in certain clinical contexts when determining whether patients are likely to respond to conservative management or require earlier surgical intervention. Future research should therefore explore whether the duration of symptom onset during standardized provocative tests could serve as a predictor of treatment response and help guide individualized treatment planning.

In conclusion, this study found no correlation between the time to symptom onset during the controlled-pressure provocative test and the severity of carpal tunnel syndrome as determined by electrodiagnostic findings. In clinical practice, positive provocative tests should continue to be used for diagnosis, but clinicians should not use the duration of symptom onset as a proxy for CTS severity. Electrodiagnostic testing remains the standard for severity grading and treatment planning.
